# Lessons Learned from Implementation of an Interferon Gamma Release Assay to Screen for Latent Tuberculosis Infection in a Large Multicenter Observational Cohort Study in Brazil

**DOI:** 10.1128/Spectrum.01163-21

**Published:** 2021-12-01

**Authors:** Allyson G. Costa, Brenda K. S. Carvalho, Mariana Araújo-Pereira, Hiochelson N. S. Ibiapina, Renata Spener-Gomes, Alexandra B. Souza, Adriano Gomes-Silva, Alice M. S. Andrade, Elisangela C. Silva, María B. Arriaga, Aline Benjamin, Michael S. Rocha, Adriana S. R. Moreira, Jamile G. Oliveira, Marina C. Figueiredo, Megan M. Turner, Betina Durovni, Solange Cavalcante, Afranio L. Kritski, Valeria C. Rolla, Timothy R. Sterling, Bruno B. Andrade, Marcelo Cordeiro-Santos, RePORT-Brazil Consortium

**Affiliations:** a Instituto de Pesquisa Clínica Carlos Borborema, Fundação de Medicina Tropical Doutor Heitor Vieira Dourado, Manaus, Brazil; b Programa de Pós-Graduação em Medicina Tropical, Universidade do Estado do Amazonas, Manaus, Brazil; c Escola de Enfermagem de Manaus, Universidade Federal do Amazonas, Manaus, Brazil; d Diretoria de Ensino e Pesquisa, Fundação Hospitalar de Hematologia e Hemoterapia do Amazonas, Manaus, Brazil; e Laboratório de Inflamação e Biomarcadores, Instituto Gonçalo Moniz, Fundação Oswaldo Cruz, Salvador, Brazil; f Faculdade de Medicina, Universidade Federal da Bahia, Salvador, Brazil; g Multinational Organization Network Sponsoring Translational and Epidemiological Research Initiative, Salvador, Brazil; h Instituto Nacional de Infectologia Evandro Chagas, Fundação Oswaldo Cruz, Rio de Janeiro, Brazil; i Instituto Brasileiro para Investigação da Tuberculose, Fundação José Silveira, Salvador, Brazil; j Programa Acadêmico de Tuberculose, Faculdade de Medicina, Universidade Federal do Rio de Janeiro, Rio de Janeiro, Brazil; k Centro de Biociências e Biotecnologia, Universidade Estadual do Norte Fluminense Darcy Ribeiro, Rio de Janeiro, Brazil; l Fundação Saúde do Estado do Rio de Janeiro, Secretaria Estadual de Saúde do Estado do Rio d Janeiro, Rio de Janeiro, Brazil; m Secretaria Municipal de Saúde do Rio de Janeiro, Rio de Janeiro, Brazil; n Division of Infectious Diseases, Department of Medicine, Vanderbilt University School of Medicine, Nashville, Tennessee, USA; o Faculdade de Medicina, Universidade Nilton Lins, Manaus, Brazil; Quest Diagnostics Nichols Institute

**Keywords:** tuberculosis, IGRA, QuantiFERON-Plus, LTBI, screening, quality control

## Abstract

The interferon gamma release assay (IGRA) has emerged as a useful tool for identifying latent tuberculosis infection (LTBI). This assay can be performed through testing platforms such as the QuantiFERON-TB Gold Plus (QFT-Plus) assay. This *in vitro* test has been incorporated into several guidelines worldwide and has recently been considered by the World Health Organization (WHO) for the diagnosis of LTBI. The possibility of systematically implementing IGRAs such as the QFT-Plus assay in centers that perform LTBI screening has been accelerated by the decreased availability of the tuberculin skin test (TST) in several countries. Nevertheless, the process to implement IGRA testing in routine clinical care has many gaps. The study utilized the expertise acquired by the laboratory teams of the Regional Prospective Observational Research in Tuberculosis (RePORT)-Brazil consortium during study protocol implementation of LTBI screening of tuberculosis (TB) close contacts. RePORT-Brazil includes clinical research sites from Brazilian cities and is the largest multicenter cohort of TB close contacts in the country to date. Operational and logistical challenges faced during IGRA implementation in all study laboratories are described, as well as the solutions that were developed and led to the successful establishment of IGRA testing in RePORT-Brazil. The descriptions of the problems identified and resolved in this study can assist laboratories implementing IGRAs, in addition to manufacturers of IGRAs providing effective technical support. This will facilitate the implementation of IGRA testing in countries with large TB burdens, such as Brazil.

**IMPORTANCE** The IGRA has emerged as a useful tool for identifying persons with LTBI. Although the implementation of IGRAs is of utmost importance, to our knowledge there is scarce information on the identification of logistical and technical challenges for systematic screening for LTBI on a large scale. Thus, the descriptions of the problems identified and resolved in this study can assist laboratories implementing IGRAs, in addition to manufacturers of IGRAs providing effective technical support. This will facilitate the implementation of IGRA testing in countries with large TB burdens, such as Brazil.

## INTRODUCTION

The World Health Organization (WHO) estimates that one-quarter of the global population is infected with Mycobacterium tuberculosis (1, 2). Most individuals exposed to M. tuberculosis who become infected are asymptomatic, and such cases are referred to as latent tuberculosis infection (LTBI). Between 5% and 10% of individuals with LTBI, if not treated with tuberculosis preventive therapy (TPT), can progress to active tuberculosis (TB) during their lifetime (3, 4). Thus, diagnosis and treatment of LTBI are critical to reduce the incidence of active TB and to control M. tuberculosis transmission.

In several countries in which TB is endemic, such as Brazil, screening for LTBI has traditionally utilized the tuberculin skin test (TST), which consists of intradermal inoculation of the TB purified protein derivative (PPD) and evaluation for cutaneous induration. However, this test has several limitations, such as false-positive reactions in persons with Mycobacterium bovis BCG vaccination or infection with nontuberculous mycobacteria and false-negative reactions in persons with immune suppression (5, 6). Additional issues include the requirement for a return visit to assess the skin for induration within 48 to 72 h after PPD inoculation and the subjective interpretation of the dermal reaction (i.e., interreader variability) (7, 8). Recent scientific advances in molecular investigations allowed the isolation of M. tuberculosis-specific antigens that drive production of interferon gamma (IFN-γ) by specific T lymphocytes, enabling the development of more specific assays based on cellular recall responses to identify LTBI (9, 10).

The IFN-γ release assay (IGRA) has emerged as a useful tool in identifying persons with LTBI. This assay can be performed through two distinct testing platforms, such as the enzyme-linked immunosorbent spot assay (ELISPOT) (T-SPOT.TB) or the QuantiFERON-TB Gold Plus (QFT-Plus) assay. These *in vitro* tests have been incorporated into several guidelines worldwide and have recently been considered by the WHO as equivalent for the diagnosis of LTBI (9, 10). Of note, both IGRA tests have advantages over TST in several aspects, including the following: (i) they do not require a follow-up visit to obtain results and (ii) they use *in vitro* stimulation of cells from the peripheral blood with M. tuberculosis-derived ESAT-6 and CFP-10 proteins, which are absent in the BCG vaccine and in most nontuberculous mycobacteria, resulting in higher specificity (11–13). The advantages of the IGRA over the TST indicate that this immunoassay may be a reliable alternative. However, the use of the IGRA results in high costs and the necessity to be carried out in sites using good clinical laboratory practice (GCLP), with well-trained technical personnel and available equipment.

The advantages of the IGRA over the TST indicate that this immunoassay may be a reliable alternative to the TST. The possibility of systematically implementing IGRAs such as QFT-Plus in centers that perform LTBI screening has been accelerated by the decreased availability of TST in several countries, including Brazil (14). Implementation of QFT-Plus in TB reference centers could facilitate screening, diagnosis, and treatment of LTBI and thereby reduce the TB burden.

Although the implementation of IGRAs is of utmost importance, to our knowledge there is scarce information on the identification of logistical and technical challenges for systematic screening for LTBI on a large scale. The present study was designed to fill this gap and provide information that would improve the effectiveness and efficiency of IGRA-based LTBI screening. The operational and logistical challenges faced during IGRA implementation in all four study laboratories are also described, as well as the solutions that were developed and led to the successful establishment of IGRA testing in RePORT-Brazil.

## RESULTS

### IGRA implementation.

**FIG 1:**
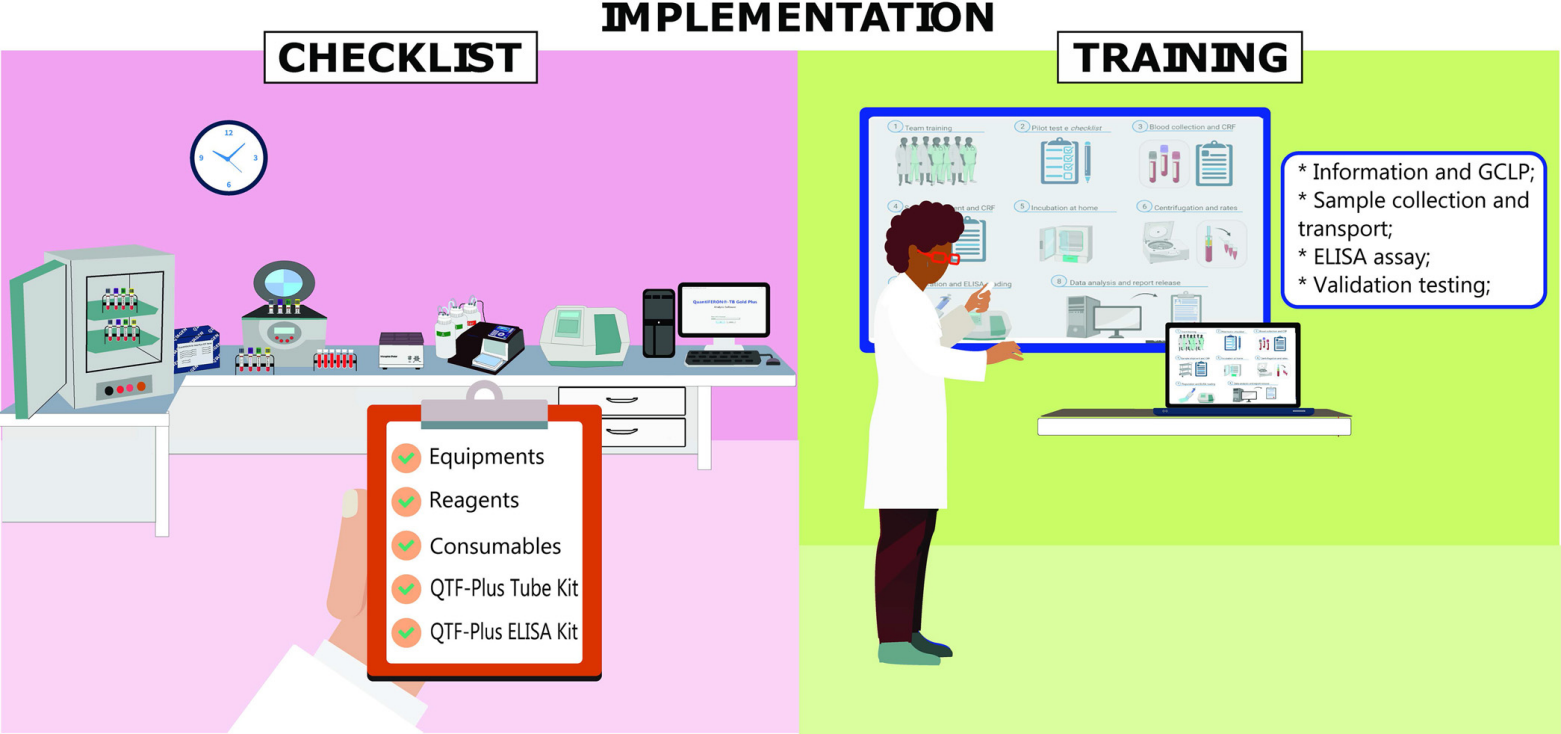
Description of IGRA/QFT-Plus implementation in the study. The first step was to check whether all equipment and reagents were available and in good condition. Then, the team was trained to perform the test following GCLP and according to the manufacturer’s recommendations.

The IGRA/QFT-Plus implementation process started with the checklist provided by Qiagen Corp. The four laboratories proceeded with the acquisition of equipment (micropipettes, 37°C incubator, centrifuge, refrigerator, −20°C freezer, microplate washer, microplate reader, and computer), reagents (ultrapure distilled water and cleaning solutions), consumables (tips, microtubes, and solution reservoirs), QFT-Plus tube kits, and QFT-Plus enzyme-linked immunosorbent assay (ELISA) kits. Subsequently, laboratory technicians took GCLP training and were trained on the steps of collecting, transporting, incubating, and processing samples for laboratory tests. In addition, QFT-Plus ELISAs were performed, and a pilot test was carried out, with the objective of including improvement strategies to initiate the study protocol (Fig. 1).

During the IGRA implementation process, several gaps were identified, even after following all of the recommendations by the manufacturer of the QFT-Plus assay. Initially, the laboratories identified problems related to acquisition of equipment, reagents, and consumables. The equipment had to be purchased through the sites, generating additional costs. Furthermore, the microplate washer had problems at the beginning of the ELISAs, requiring corrective maintenance. QFT-Plus tube kits and QFT-Plus ELISA kits were imported, which generated logistical problems regarding delivery to the sites. To resolve this problem, delivery of the QFT-Plus tubes and ELISA kits was directed to one of the clinical sites in Rio de Janeiro, which then distributed the supplies to the other laboratories.

### IGRA under routine conditions.

**FIG 2:**
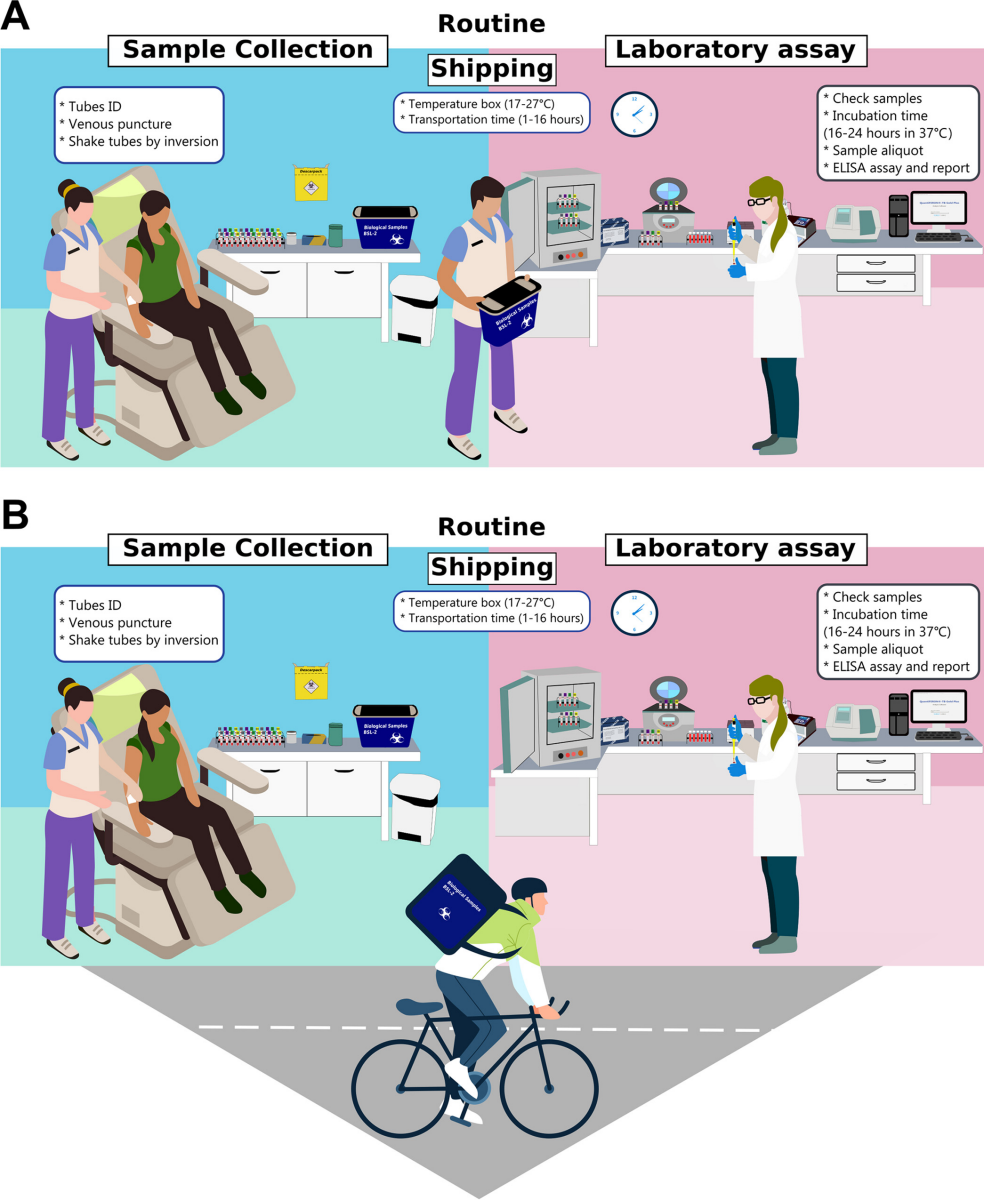
Two different conditions for IGRA/QFT-Plus sample collection and processing in the study. (A) Setup A, utilized by sites 1 and 2 and characterized by performing the collection and processing of samples in the same place, without a vehicle. (B) Setup B, utilized by sites 3, 4, and 5 and characterized by performing the collection and processing of samples in different places, with transportation of the samples by vehicle.

The five clinical sites started recruiting patients and collecting samples under routine conditions. Prior to starting, the collecting station was organized, accounting for the environment and the tubes to be used in the QFT-Plus test. These tubes had to be stored between 4°C and 25°C and taken out for immediate use only. In addition, the tubes had to be collected in a specific order, following the manufacturer's recommendations, with the nil tube being collected first, followed by the TB1, TB2, and mitogen tubes. The tubes containing the samples went through a homogenization process that consisted of several inversions, in which all of the biological material had to come into contact with the inner surface of the tube (L-motion with exactly 10 inversions). Finally, QFT-Plus tubes were packed in boxes with temperatures ranging from 17°C to 27°C for transfer to the processing laboratory (Fig. 2).

After collection at the clinical sites, samples were transported to the four RePORT-Brazil laboratories in two different settings, as follows. Setup A (pertaining to sites 1 and 2) was characterized by performing the collection and processing of samples in the same place, with the collection room and laboratory within the same site. Thus, the study staff responsible for collecting and organizing the samples could transport the samples to the laboratory without requiring a vehicle (bicycle, motorcycle, or car) (Fig. 2A). Setup B (pertaining to sites 3, 4, and 5) was characterized by performing the collection and processing of samples in different places, with the need for transportation to the laboratory and thus greater demand for time, organization, and attention so that there was no excessive tube vibration. This setup required a vehicle (bicycle, motorcycle, or car) to transport samples (Fig. 2B).

Upon arrival at the laboratories, samples had to pass a quality check regarding transport time, temperature, tube identification (ID), volume, and the presence of hemolysis or clots. Finally, the samples were processed and incubated at 37°C for 20 h, aliquots were prepared, and the plasma samples were frozen at −20°C until the QFT-Plus ELISA was performed.

### IGRA results under routine conditions in two settings.

**FIG 3:**
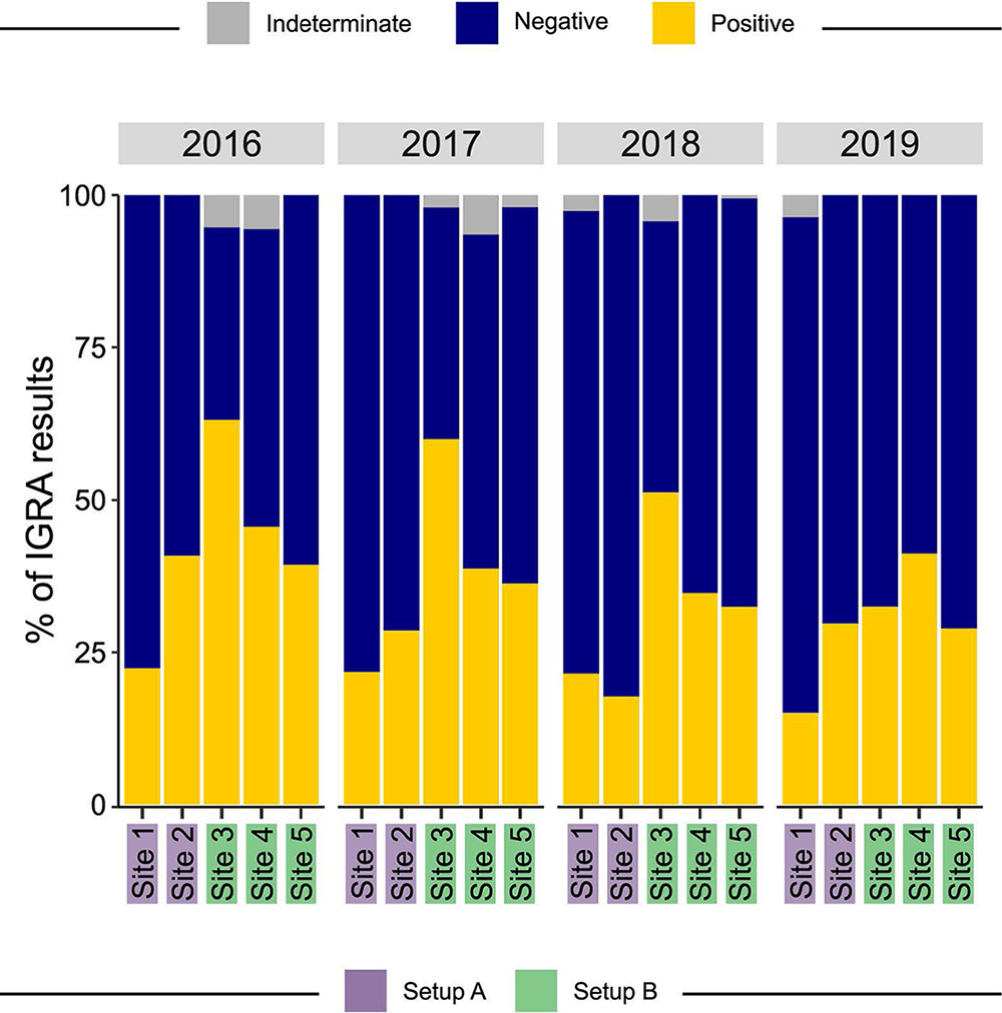
Frequencies of IGRA/QFT-Plus results in each setup and site, stratified by year of the study. The sites are grouped as setup A (sites 1 and 2) and setup B (sites 3, 4, and 5).

Figure 3 summarizes the IGRA/QFT-Plus results under routine conditions in setups A and B at sites 1 to 5 during the 4-year study period (2016 to 2019). These results were obtained after the QFT-Plus ELISAs were performed by the teams at the sites. For this, the aliquots containing the samples were thawed and used only once. In addition to the recommendations indicated by the manufacturer, the laboratories underwent quality control with the results being validated by an external laboratory. Setup B had a higher percentage of indeterminate results, mainly in the first years of implementation of QFT-Plus testing. In addition, the number of samples changed over time for each setup, as follows: 2016: setup A, 190 samples; setup B, 173 samples; 2017: setup A, 366 samples; setup B, 258 samples; 2018: setup A, 505 samples; setup B, 471 samples; 2019: setup A, 570 samples; setup B, 333 samples. Also note that, over the years of implementation, the indeterminate percentage tended to decrease. This could be associated with the learning process for site personnel. In addition, higher levels of undetermined results were observed in setup A in 2018 and 2019, specifically at site 1, which could be attributed to a greater number of tests performed in this setup and site.

### Nonconformities in IGRA testing.

**FIG 4:**
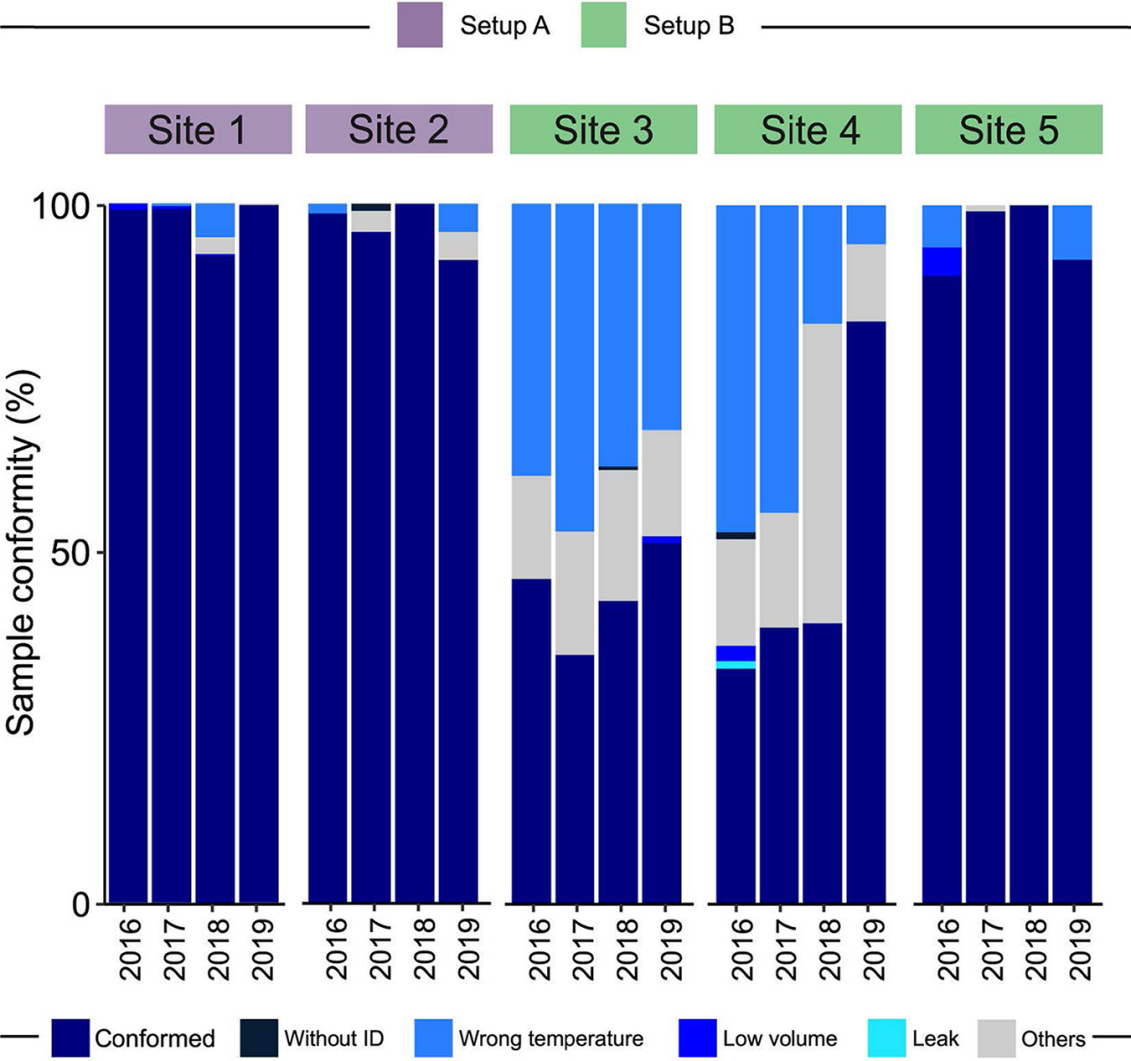
Frequencies of conformities and nonconformities of samples in each setup and site, stratified by year of the study. The sites are grouped as setup A (sites 1 and 2) and setup B (sites 3, 4, and 5).

During the QFT-Plus implementation process, several problems were detected, generating nonconformities, such as samples without an ID, transport with temperatures outside the established standard (temperature deviation), sample leakage, and other issues (e.g., transport box change, coagulated samples, or a nonstandard set of transport conditions). The proportions of nonconformities are shown in Figure 4.

The temperature deviation and other nonconformities were noted particularly among setup B sites (see Table S1 in the supplemental material), compared to setup A sites, in all years analyzed (*P* < 0.001). These results were likely due to conditions related to the collection and transportation of samples, since setup B required sample transport by vehicle. In addition, these gaps generated learning opportunities for the teams, which over the years of implementation decreased the proportions of reported nonconformities. Of note, setup B also presented significantly higher occurrences of transport box change, coagulated samples, and lack of minimum transport conditions, characterized in Table S1 as “Others” (*P* < 0.001).

### Temperature deviation was the main nonconformity in IGRAs.

**FIG 5:**
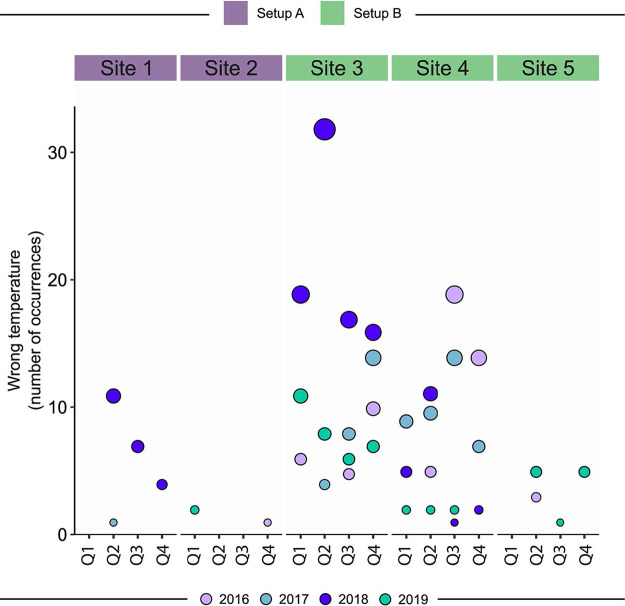
Number of occurrences of the nonconformity temperature deviation in each setup and site, stratified by quarter (Q) and year of the study. The sites are grouped as setup A (sites 1 and 2) and setup B (sites 3, 4, and 5). The color of the circles indicates the year of nonconformity recorded, and the size is proportional to the number of occurrences.

Temperature deviation was the main nonconformity identified in our study. When the occurrence was observed over the quarters of the evaluated years, deviation occurred mainly in the months with the highest temperatures at the sites, such as spring and summer (third, fourth, and first quarters) (Fig. 5). There was a decrease in this nonconformity over the years of the study period.

### Dynamics of the time and temperature between collection and processing of IGRA samples.

The distance between the collection site and the sample-processing laboratory directly influenced the time and the temperature variation during transportation (Table 1). Setup A showed a significantly shorter time between sending and receiving the samples in all studied years (*P* < 0.001), influencing the temperature variation, which was also significantly less in comparison to setup B (*P* < 0.001).

**TABLE 1:** Time and temperature quality control measurements by year in the study period

Parameter and year	Setup A^*a*^	Setup B	*P* ^*b*^
Time between sending and receiving (median [IQR]) (min)			
2016	55.8 (20.1–75.1)	140.0 (102.0–176.0)	<0.001
2017	58.7 (22.2–82.8)	121.0 (83.5–155.0)	<0.001
2018	61.8 (25.1–90.1)	89.0 (29.0–164.0)	<0.001
2019	49.5 (20.1–66.1)	135.0 (96.0–171.0)	<0.001
Temp for sending samples (median [IQR]) (°C)			
2016	19.6 (18.1–21.2)	17.8 (16.0–19.1)	<0.001
2017	21.9 (19.6–24.1)	18.0 (16.0–19.5)	<0.001
2018	21.5 (19.3–23.8)	18.3 (16.8–19.8)	<0.001
2019	20.6 (19.0–22.2)	18.1 (16.6–19.2)	<0.001
Temp for receiving samples (median [IQR]) (°C)			
2016	20.1 (18.8–21.4)	19.6 (17.9–21.6)	<0.001
2017	22.1 (20.6–24.1)	20.7 (19.5–22.9)	<0.001
2018	21.9 (20.0–23.9)	20.4 (18.6–22.9)	<0.001
2019	20.7 (19.5–22.1)	20.5 (18.9–22.6)	<0.001
Temp variation (receiving vs sending) (median [IQR]) (°C)			
2016	0.54 (0.0–0.8)	1.82 (0.0–3.7)	<0.001
2017	0.26 (0.0–0.1)	2.72 (1.2–4.1)	<0.001
2018	0.37 (0.0–0.4)	2.06 (0.9–4.1)	<0.001
2019	0.15 (0.0–0.2)	2.35 (1.1–4.3)	<0.001

a Setup A contains sites 1 and 2 and is characterized by performing the collection and processing of samples in the same place. Setup B contains sites 3, 4, and 5 and is characterized by performing the collection and processing of samples in different places. The numbers of samples for each setup changed over time, as follows: 2016: setup A, 190 samples; setup B, 173 samples; 2017: setup A, 366 samples; setup B, 258 samples; 2018: setup A, 505 samples; setup B, 471 samples; 2019: setup A, 570 samples; setup B, 333 samples.

b Data were compared between the setups using the Mann-Whitney U test. All *P* values indicate statistical significance.

**FIG 6:**
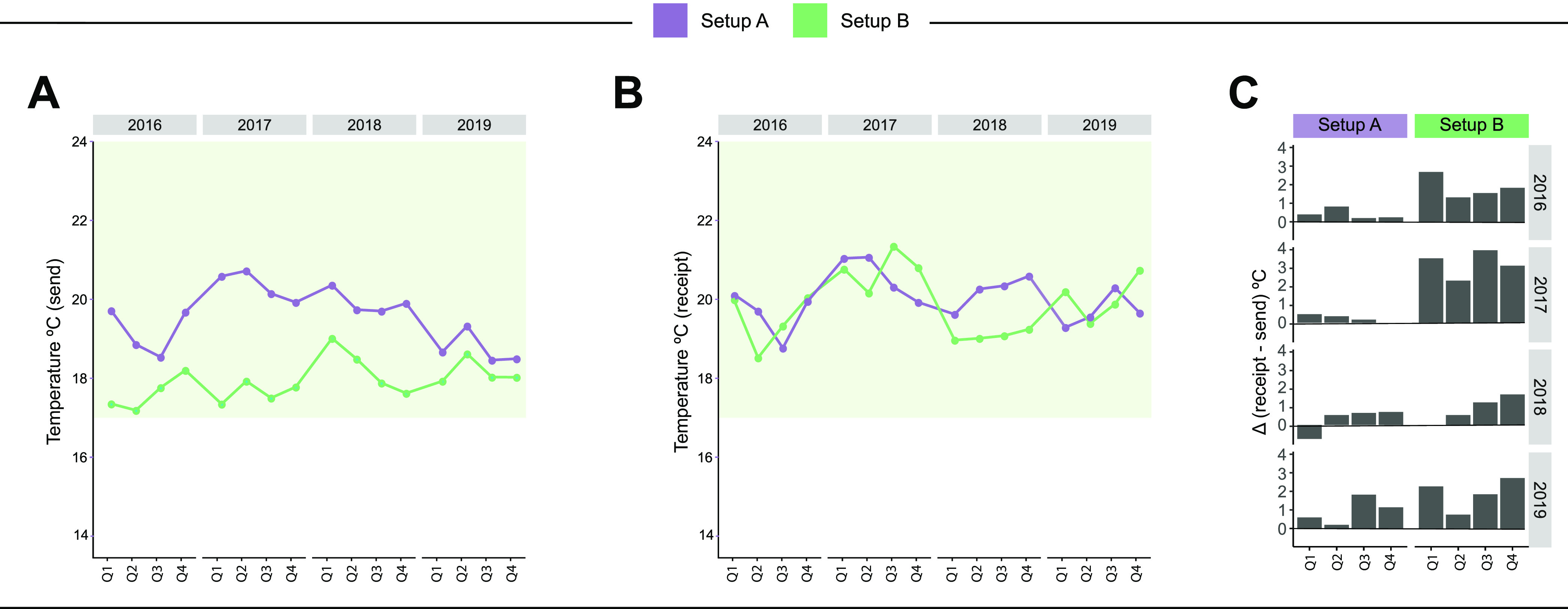
Dynamics of the temperature at the time of shipment and receipt of study samples and the temperature variation over time in each setup, stratified by quarter (Q) and year during the study period. (A) Average temperature of sending samples calculated by quarter and year in each setup. (B) Average temperature of receiving samples calculated by quarter and year in each setup. (C) The difference between receiving and sending temperatures (Δ) was calculated for each quarter and year in each setup. Purple lines indicate setup A, and green lines indicate setup B. The light green blocks indicate the limit accepted by the IGRA test manufacturer as acceptable for the storage and handling of the samples (17°C to 27°C).

Setup A had higher temperatures at the time of specimen shipment, compared to setup B, but the two setups had similar temperatures at the time of specimen receipt/arrival. (Fig. 6A and B). Although within the established standard, setup A had significantly higher temperatures at the time of both shipment and receipt. Despite this, the temperature variation was significantly greater in setup B (Fig. 6C). This could also be seen when the data were analyzed by site (see Fig. S1).

Over the years of implementation, the variation in time between sending and receiving samples tended to decrease until 2018, and then there was an increase in the variation in 2019. The decrease in time variation was possibly because study and transport (collection, sending, and processing) became more efficient with the implementation of the test at the sites. Although we observed this increase in 2019, it is noteworthy that there was an increase in the number of samples analyzed that year.

**FIG 7:**
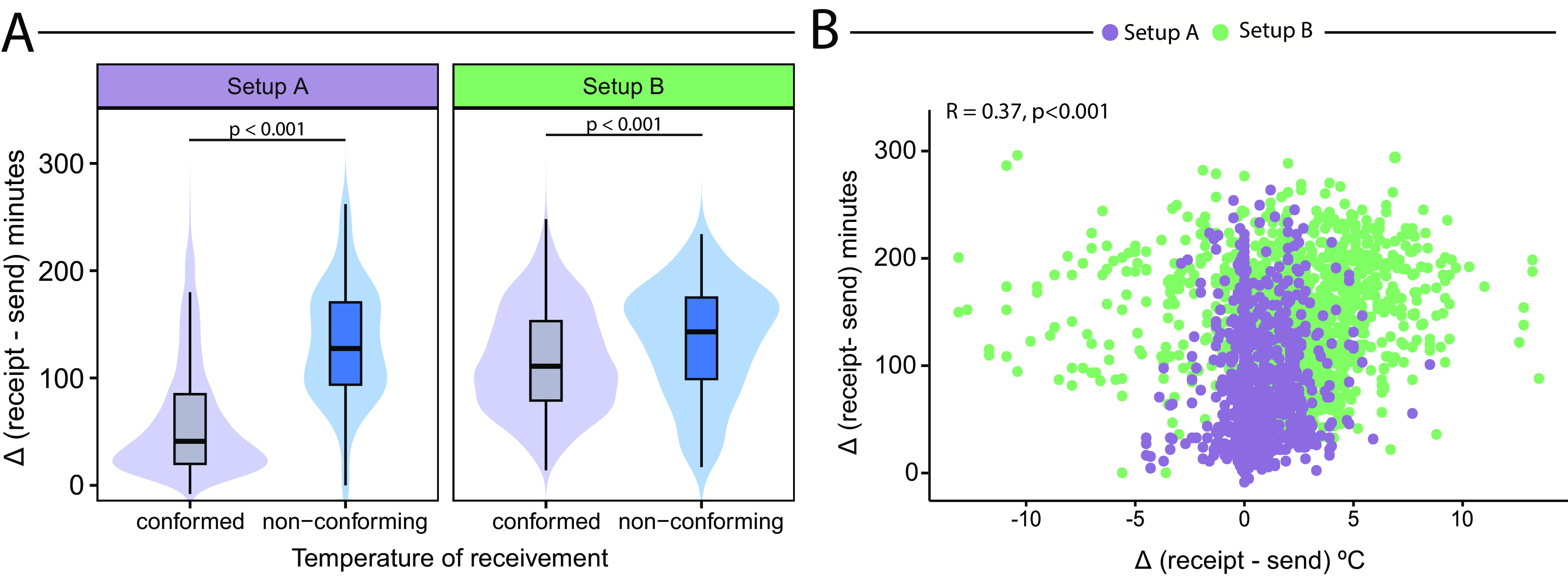
Correlation between the variation of receiving versus sending time and sample temperature. (A) Dynamics of the presence of nonconformity in the sample receiving temperature versus the time variation (variation of receiving versus sending). Nonconformity was defined when the receiving temperature was <17°C or >27°C. (B) Correlation between temperature variation and time variation.

Finally, we performed categorical analysis of the presence of nonconformity in the sample receiving temperature versus the variation in receiving versus sending time (Fig. 7A). Significant differences between setup A and setup B were observed when the time variation was compared with the presence of the nonconformity analyzed, showing that a greater time variation could lead to a nonconformity in temperature. Importantly, it was noted that the variation in the time of the samples conforming in setup A was smaller. In addition, the numbers of samples in conformity and nonconformity for each setup were different, as follows: setup A: conformity, 1,541 samples (91.7%); nonconformity, 140 samples (8.3%); setup B: conformity, 1,077 samples (88.7%); nonconformity, 137 samples (11.3%). Lastly, a correlation analysis was performed between the variation in receiving *versus* sending time and the variation in temperature. In this analysis, it was possible to note that the temperature variation was directly correlated with the time variation (*r* = 0.37, *P* < 0.001) and that this variation was particularly seen in setup B (Fig. 7B).

## DISCUSSION

In the IGRA implementation process, gaps were identified mainly in the preanalytical phase of the QFT-Plus assay. The training of laboratory technicians without experience in sample collection, transporting, and processing for ELISAs was straightforward and the learning curve was short, despite preanalytical errors identified after the start of the study. In addition to the recommendations indicated by the manufacturer, the laboratories underwent quality control with the results being validated by an external laboratory and annual retraining on sample collection, transport, and processing steps. Preanalytical errors have important implications for the reproducibility and accuracy of IGRAs, indicating the need to standardize the preanalytical steps, as shown previously (15). The sites standardized the preventive maintenance process for the equipment, to minimize problems. The delivery of the QFT-Plus tubes and ELISA kits was directed to one of the clinical sites, which then distributed the supplies to the other laboratories, mitigating import and logistic issues. The steps of collection, transport, and processing of the samples started to be monitored via case report forms (CRFs). The lessons learned at this stage were important to create mechanisms for tracking nonconformities, It is noteworthy that the use of CRFs is indicated for monitoring nonconformities in clinical trials; however, they can also be used under routine laboratory conditions (16).

The sample collection, transport, and processing settings (setups A and B) in the study influenced the results of the QFT-Plus assay. Setup B had a higher percentage of indeterminate and positive results, probably due to the longer time between the collection and processing of the samples, in addition to other variables evaluated here (e.g., temperature of transport, ID of the tubes, and packaging of the samples). It is important to ensure that, in the analytical phase, the laboratory technicians involved in these steps are comfortable with performing the QFT-Plus assay, including simple tasks such as controlling the temperature of the shipping boxes, identifying nonconformities, minimizing errors, and identifying problems in the results generated. It is recommended to include in the QFT-Plus implementation planning a period of adaptation and short retraining directed at the critical stages of sample collection, transport, and processing, with the aim of gradually minimizing or eliminating nonconformities and preanalytical errors. This learning can assist in the quality control of test results and performance, since the reproducibility of IGRAs can be influenced by these factors (17).

Biological samples can have different performance or results with respect to the quantity of analytes used and, based on this, we must seek to identify points that cause variation of test results. The laboratory routine can have different components that can cause variability in results, such as different processing rates, variations in processing rates throughout the month, and variations of factors related to the environment (such as temperature) throughout the year (18).

The reproducibility of the test results depends directly on the training of the team, since variation in the operator for the collection, homogenization, and performance of the test can impact the results. Therefore, the standardization of quality control in the clinical and laboratory spheres is essential so that there are no significant effects on the result. In addition, structural variations between different laboratories can influence the proportions of indeterminate results (17, 19).

With regard to immunological molecules that can be released and consumed quickly *in vitro*, delays in the start of the incubation of study samples can interfere with the quantification of IFN-γ levels, leading to a decrease of up to 0.24 IU/ml after 6 h (15, 17). Different incubation times, without a specific pattern, can possibly influence test results, since in our study the distance between the collection site and the sample-processing laboratory directly influenced the time and the temperature variation during transportation. Therefore, it is important to follow the manufacturer's guidelines (17, 19). Furthermore, the required final volume of biological sample must always be used, and it is not possible to use volumes smaller or larger than that recommended for the test. Due to the amount of “biological stimulus” available per tube, the amount of IFN-γ released at the end of the test may be affected by variation in the sample volume used (17, 20, 21).

The expansion of the use of IGRAs, such as QFT-Plus, must be well planned, with negotiations with the manufacturer regarding the logistics of delivery of the kits in areas with difficult access. In addition, the sample collection, transport, and processing settings must be evaluated, with the aim of mitigating errors that may interfere with the test results. Training of laboratory technicians is extremely important, and regular training develops a sense of responsibility toward reporting nonconformities and maintaining data quality after the initial implementation. In addition, we recommend including the use of CRFs for the sample collection, transport, and processing steps, to monitor mainly the time and temperature variations, as these directly influence the IGRA test results, as well as quality control, with the results being validated by an external laboratory, and annual retraining on implementation steps. Finally, we think that some of the problems identified in this study can assist laboratories wishing to implement IGRA testing, in addition to manufacturers of IGRAs providing effective technical support. These findings may facilitate the implementation of IGRA testing in countries with large TB burdens, such as Brazil.

## MATERIALS AND METHODS

### Study design and laboratory sites.

The present investigation was an implementation study performed within a multicenter cohort study (RePORT-Brazil) between 2016 and 2019. It was conducted in five research centers located in four Brazilian cities, namely, Fundação de Medicina Tropical Dr. Heitor Vieira Dourado (FMT-HVD) in Manaus-Amazonas, Instituto Brasileiro para Investigação da Tuberculose (IBIT) in Salvador-Bahia, Secretaria Municipal de Saúde de Duque de Caxias (SMS-DC) in Duque de Caxias-Rio de Janeiro, and Instituto Nacional de Infectologia Evandro Chagas (INI) and Clínica da Família Rinaldo Delamare, Secretaria Municipal de Saúde do Rio de Janeiro (SMS-RJ), in Rio de Janeiro (22). The five health centers are located in three distinct regions of Brazil, with similar climate conditions (equatorial and tropical), temperature, and humidity, ranging from 19.9°C to 26.4°C and from 77.2% to 85.1%, respectively (23, 24).

### Maintenance and biosafety of laboratory sites.

Standard operating procedure (SOP), good clinical practice (GCP), GCLP, and other trainings were carried out by the project staff prior to initiation of the study. In addition, all sites provided up-to-date equipment maintenance certifications to ensure test quality and to minimize risks for laboratory technicians who processed biological materials daily.

### QFT-Plus assays.

Initially, the QFT-TB Gold In-Tube (QFT-GIT) assay was implemented in the laboratory routine of the RePORT-Brazil consortium; subsequently, it was replaced by the QFT-Plus assay. Every laboratory received training on sample collection and processing by the Qiagen Corp. (Chatsworth, CA, USA). Figure S2 in the supplemental material summarizes the QFT-Plus steps. Briefly, venous blood for testing was collected in four tubes (nil, TB1, TB2, and mitogen) and incubated at 37°C for 20 h. After incubation, samples were stored at −20°C until the ELISA was performed within 2 weeks. IFN-γ levels (international units [IU] per milliliter) were quantified with a 4-point standard curve. QFT-Plus analysis software was used to generate the results according to the manufacturer’s recommendations (25). The software performed a quality control assessment of the assay, generated a standard curve, and provided quantitative (IU per milliliter) and qualitative (positive, negative, or indeterminate) results.

### Laboratory data on the QFT-Plus implementation process.

Laboratory information from the QFT-Plus implementation process was evaluated by the teams from all RePORT-Brazil laboratories. Data were obtained from team training, equipment maintenance, and preanalytical evaluations, such as type of sample collection, place of sample collection and processing, tube ID, transport quality control (type of transport, time, and temperature of the samples), presence of hemolysis or clots, and volume of the samples, as well as qualitative results (numbers of positive, negative, and indeterminate test results). All data were entered into the Research Electronic Data Capture (REDCap) platform, reviewed by data managers for quality control, and subsequently approved for the study analyses.

### Descriptive and statistical analyses.

Descriptive analyses were performed to characterize the study laboratories and quality control measurements. Categorical variables were displayed as frequency and percentages and compared using a two-sided Pearson's chi-square test (Yate’s correction) or the Fisher's two-tailed test in 2 × 3 or 2 × 2 tables, respectively. Continuous variables were displayed as median and interquartile range (IQR) and tested for Gaussian distribution using the D’Agostino-Pearson test. Comparisons of values between two groups of data were performed using the Mann-Whitney U test. The Spearman rank correlation test was carried out to assess relationships between variables. Data analyses were performed using R (version 3.6.3) and the Hmisc (version 4.4.1), compareGroups (version 4.4.3), ggplot2 (version 3.3.2), and ggcorrplot (version 0.1.3) R packages. All analyses were prespecified. Differences with *P* values of <0.05 were considered statistically significant.

### Data availability.

The original contributions presented in the study are included in the supplemental material, and further inquiries can be directed to the corresponding author.
