# Supplementary material for: Lessons Learned from Implementation of an Interferon Gamma Release Assay to Screen for Latent Tuberculosis Infection in a Large Multicenter Observational Cohort Study in Brazil
Source: Microbiol Spectr. 2021 Dec 1;9(3):e01163-21. doi: 10.1128/Spectrum.01163-21 (PMC8635161; doi:10.1128/Spectrum.01163-21)
Supplement: SUPPLEMENTAL FILE 1 — Supplemental material. Download SPECTRUM01163-21_Supp_1_seq11.pdf, PDF file, 0.5 MB [file spectrum01163-21_supp_1_seq11.pdf]

Supplemental Material 1

Table S1: Non-conformities stratified by year in period of study.

Non-Conformities	Year	Setup A*	Setup B	p-value#
Without ID, n (%)£	2016¥	0 (0)	1 (0.6)	0.96
	2017	0 (0)	1 (0.4)	1.00
	2018	0 (0)	1 (0.2)	0.97
	2019	0 (0)	0 (0)	1.00
Low volume, n (%)	2016	1 (0.5)	4 (2.3)	0.31
	2017	1 (0.3)	0 (0)	1.00
	2018	1 (0.2)	0 (0)	1.00
	2019	0 (0)	1 (0.3)	0.78
Wrong temperature, n (%)	2016	1 (0.5)	67 (38.7)	***< 0.001***
	2017	1 (0.3)	77 (29.8)	***< 0.001***
	2018	20 (4.0)	92 (19.5)	***< 0.001***
	2019	2 (0.4)	51 (15.3)	***< 0.001***
Other§, n (%)	2016	1 (0.5)	22 ((12.7)	***< 0.001***
	2017	3 (0.8)	30 (11.6)	***< 0.001***
	2018	10 (2.0)	84 (17.8)	***< 0.001***
	2019	3 (0.5)	28 (8.4)	***< 0.001***

*Setup A: contains Site 1 and 2, characterized by performing the collection and processing of samples in the same place. Setup B: contains Site 3, 4 and 5. It is characterized by performing the collection and processing of samples in distinct place; #Data were compared between the Pearson's χ^2 test. Bold and italic font indicates statistical significance. ¥The number of samples changes overtime for each Setup, as follow: 2016 (A = 190; B = 173), 2017 (A = 366; B = 258), 2018 (A = 505; B = 471), 2019 (A = 570; B = 333). £Data are shown as number (n) and frequency (percentage). §Other: Transport Box Change, coagulated samples or without minimum transport conditions.

Supplemental Material 2

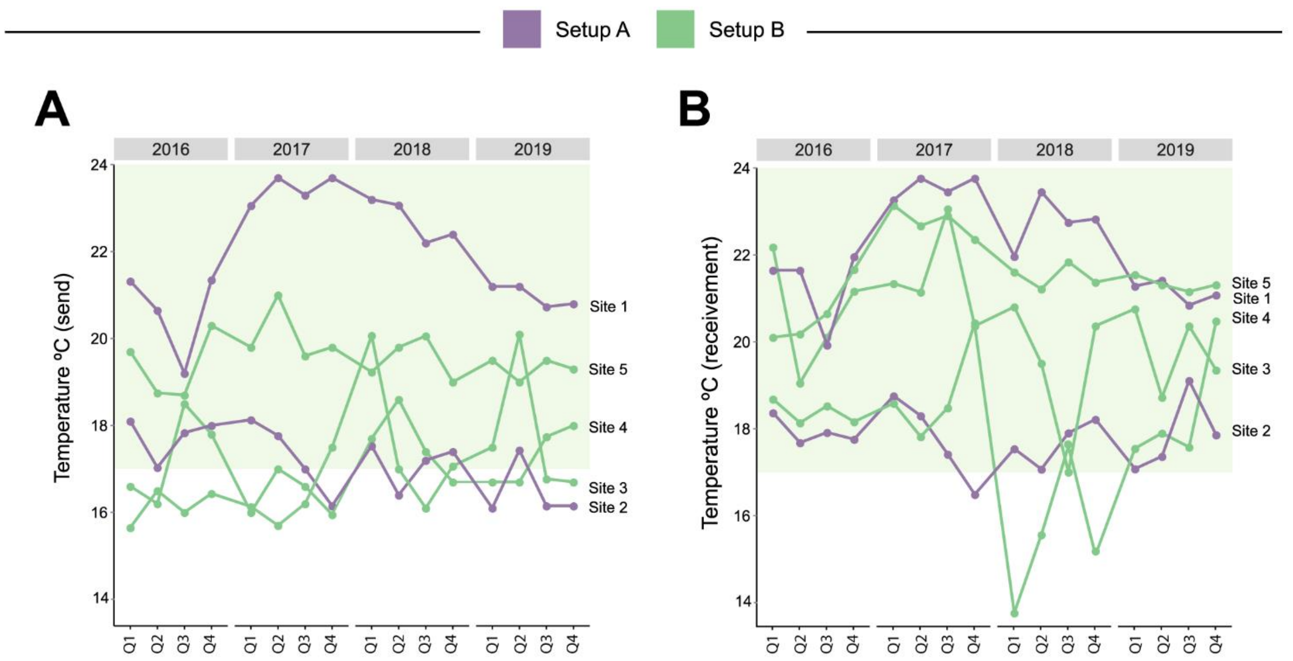

Figure S1: Dynamics of the temperature at the time of shipment and receipt of samples, and the delta temperature variation over time in each Setup and Site stratified by trimester and year during the study period. A) Average Temperature (°C) of sending samples calculated by trimester and year in each Setup and Site. B) Average Temperature (°C) of receiving samples calculated by trimester and year in each Setup and Site. Purple lines indicate Setup A and green lines indicate Setup B. The light green block indicates the limit accepted by the IGRA test manufacturer as acceptable for the storage and handling of the samples (17-27ºC).

Supplemental Material 3

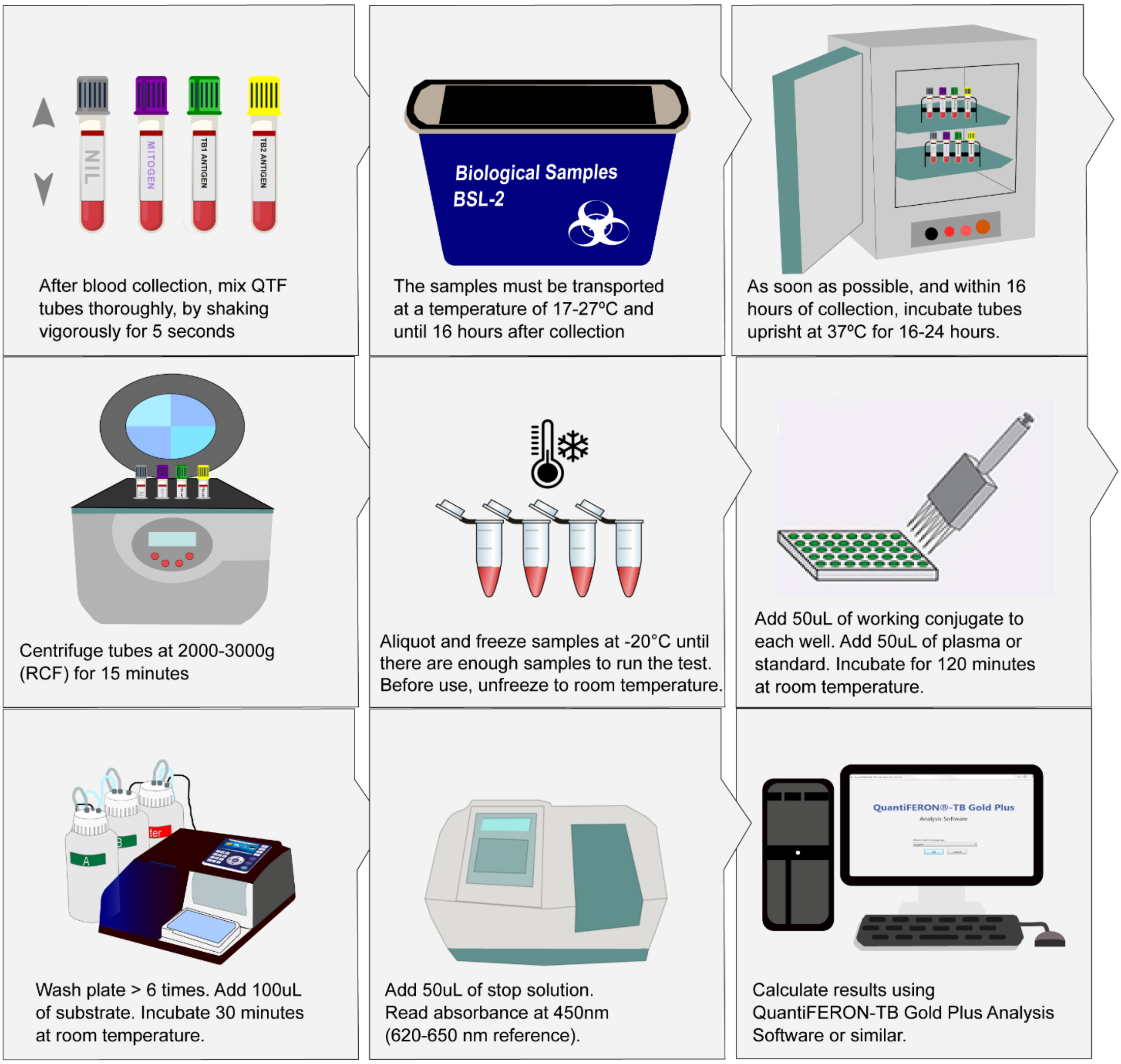

Figure S2: Steps of the QFT®-Plus in the study setting.
